# OMI/NOMI: Time for a New Classification of Acute Myocardial Infarction

**DOI:** 10.3390/jcm13175201

**Published:** 2024-09-02

**Authors:** Martiola Kola, Naltin Shuka, Harvey Pendell Meyers, Elizana Zaimi (Petrela), Stephen W. Smith

**Affiliations:** 1 Cardiology, University Hospital Center Mother Teresa, 1001 Tirana, Albania; 2 Cardiovascular Medicine, University Hospital Center Mother Teresa, 1001 Tirana, Albania; naltinshuka@yahoo.com; 3 Emergency Medicine, Carolinas Medical Center, Charlotte, NC 28203, USA; pendellmeyers@gmail.com; 4 Public Health, Faculty of Medicine, University of Medicine, 1005 Tirana, Albania; elapetrela@yahoo.com; 5 Emergency Medicine, Hennepin Healthcare, Minneapolis, MN 55415, USA; smith253@gmail.com

**Keywords:** OMI, NOMI, STEMI, NSTEMI, PCI, PPCI

## Abstract

Forty percent of patients with acute coronary occlusion myocardial infarction (OMI) do not present with STEMI criteria, which delays their treatment and increases morbidity and mortality. The need to identify these patients promptly is crucial, and this sets the stage for the proposed reclassification. Many of these patients can be identified by other ECG and clinical features. **Background/Objectives**: We sought to evaluate cases of STEMI and NSTEMI that result in OMI. Additionally, we focused on the consequences of delayed revascularization in NSTEMI patients with acute coronary occlusion (NSTEMI-OMI). **Methods**: The study is a retrospective analysis conducted on 334 patients who underwent coronary angiography for acute coronary syndrome at UHC “Mother Teresa”, Tirana, Albania, during January–May 2023. “OMI was defined as an acute culprit lesion with TIMI 0–2 flow, or an acute culprit lesion with TIMI 3 flow intervened upon and with highly elevated troponin (cTnI > 10.0 ng/mL, hs-cTnI > 5000 ng/L)”. The presence or absence of STEMI criteria were determined in the final diagnosis written on the chart by a cardiologist using the third universal definition of MI. Ejection fraction (EF), total ischemia time, length of stay, and complications were compared between groups. Mechanical complications include acute ventricular failure, cardiogenic shock, rupture of the interventricular septum, rupture of the free wall, rupture of the papillary muscle, and pericarditis. Electrical complications include ventricular arrhythmias, supraventricular arrhythmias, and atrioventricular and interventricular blocks. **Results**: There were 334 patients included, 98 (29.3%) of whom were NSTEMI-OMI patients. Ninety-six patients (40%) of OMI patients did not fulfill the STEMI criteria. Only 11 patients (11%) of STEMI(−)OMI had PCI performed within the first 12 h vs. 76 patients (77%) with STEMI(+)OMI, *p* < 0.001. There was no difference in the percent of patients requiring PCI between the STEMI(+)OMI 98 patients (93%) and STEMI(−)OMI 87 patients (89%) (*p* = 0.496). The overall in-hospital mortality was 19 patients (5.7%), with subgroup mortality of 14 patients (4.2%) with STEMI(+)OMI, 2 patients (0.6%) with STEMI(+) NOMI, and 3 patients (0.9%) with STEMI(−)OMI, 0% STEMI(−)NOMI, (*p* = 0.013). Patients with mechanical complications included 67 patients (46.8%) with STEMI(+)OMI and 45 patients (46.4%) with STEMI(−)OMI. In addition, 26 patients (18.5%) with STEMI(+)OMI and 13 patients (13.1%) with STEMI(−)OMI developed electrical complications. **Conclusions**: STEMI(−)OMI patients had significant delays in catheterization, yet had angiographic findings, rates of PCI, and complications similar to STEMI(+)OMI. These data add further support to refocusing the paradigm of acute MI to improve recognition and rapid reperfusion of all OMIs, rather than only those with STEMI criteria.

## 1. Introduction

Since “time is myocardium”, rapid reperfusion of an acute coronary occlusion (ACO) is required in order to salvage ischemic myocardium before irreversible infarction leads to increased morbidity and mortality.

For approximately two decades, the patients who were believed to benefit from emergent revascularization have been those presenting with ST elevation on the ECG, since ST elevation has been assumed to be an accurate surrogate for ACO. Thus, the dichotomy of ST elevation myocardial infarction (STEMI) vs. non-STEMI has guided the use of emergency revascularization.

Recently, there has been increasing emphasis on STEMI equivalents, which are associated with high risk. Immediate revascularization in these cases has the potential to significantly improve patient outcomes [1,2].

We know that only 43% of acute coronary occlusion MI (OMI) fulfills the STEMI millimeter (mm) criteria [3]. However, NSTEMI patients with delayed treatment of OMI have almost double mortality compared to NSTEMI patients without OMI [4]. Therefore, we support the idea of expanding the population eligible for emergent reperfusion therapy to any OMI, not only those whose ECGs manifest STEMI mm criteria. Conversely, focusing on the actual outcome of interest will also allow further research aimed at reducing false positive STEMI criteria activations [3].

The idea that all patients with an underlying ACO without collateral circulation, regardless of ECG findings, benefit from emergent revascularization has face validity, and has been recently acknowledged in the 2022 American College of Cardiology guidelines [5]. There are ECG patterns beyond ST-segment elevation mm criteria that are indicative of ACO, but, unfortunately, the “STEMI criteria” model restricts our minds and limits us so that we only recognize and emergently intervene in the STEMI subset of OMI [6].

Therefore, in 2018, the new OMI paradigm was conceived to replace STEMI/NSTEMI [7]. Blinded physicians with special expertise in OMI ECG findings demonstrated high accuracy in diagnosing OMI using the ECG, with sensitivity more than double the STEMI criteria at equal specificity [8].

## 2. Materials and Methods

The study purpose: We sought to evaluate STEMI/NSTEMI cases that result in OMI and the consequences of delayed revascularization immediately among NSTEMI patients who have ACO (NSTEMI-OMI).

Study objectives: 1. Identify the percentage of OMI patients who do not fulfill the STEMI criteria. 2. Compare the total ischemic time in each of the groups according to STEMI criteria and actual OMI outcome. 3. Compare rates of intervention, ejection fraction (EF), complications, and hospital day stay in each of these groups.

Study type: The study was performed via retrospective chart review.

**Figure 1:**
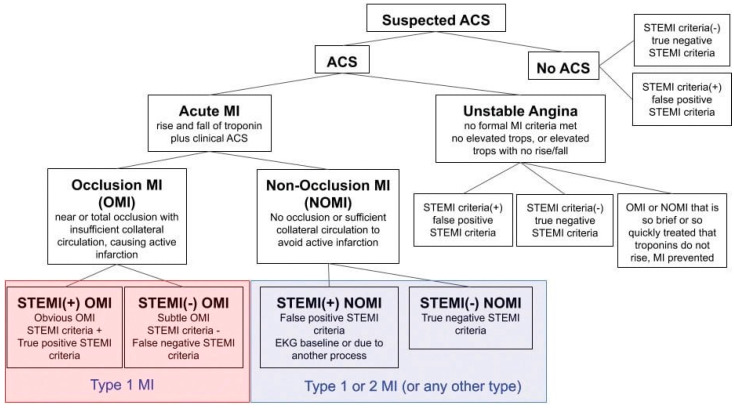
Adapted from “What Is Occlusion Myocardial Infarction (OMI)?” Powerful Medical [10]. ACS = acute coronary syndrome, acute MI = acute myocardial infarction, STEMI = ST-elevation myocardial infarction, NSTEMI = non-ST-elevation myocardial infarction, OMI = occlusion myocardial infarction, NOMI = non-occlusion myocardial infarction, MI = myocardial infarction, tops = troponins.

Data Collection: A retrospective analysis was conducted on 334 patients who underwent coronary angiography for acute coronary syndrome at UHC “Mother Teresa”, Tirana, Albania, in January–May 2023. All patients were enrolled consecutively from cardiology departments I and II and cardiology ICU in UHC “Mother Teresa” Tirana, Albania. Data such as patients’ demographic data and comorbidities, total ischemic time (the time from the onset of symptoms to the cath lab), hospital length of stay, ejection fraction, complications, and death cases were obtained from the medical records. Angiographic and relevant treatment data were collected from the standard coronary angiography registry and report, which is currently used in the cath lab, UHC “Mother Teresa” Tirana, Albania. Diagnosis of OMI vs. NOMI was determined by angiography (“confirmed OMI”). We separated patients in four groups: STEMI(+)NOMI, STEMI(−)NOMI, STEMI(−)OMI, and STEMI(+)OMI. STEMI(+) or (−) refers to cases where the criteria for ST-segment elevation myocardial infarction (STEMI) are met (+) or not (−) [Figure 1]. “OMI was defined as an acute culprit lesion with TIMI 0–2 flow, or an acute culprit lesion with TIMI 3 flow intervened upon and with highly elevated troponin (cTnI > 10.0 ng/mL, hs-cTnI > 5000 ng/L).”. The presence or absence of STEMI criteria was determined in the final diagnosis written on the chart by the cardiologist according to the third universal definition of MI [9].

Inclusion criteria: 1. Patients with a diagnosis of STEMI or NSTEMI. 2. Patients from departments I and II of cardiology and cardiology ICU who underwent coronary angiography in UHC “Mother Teresa” Tirana, Albania.

Exclusionary criteria: 1. Patients who underwent coronary angiography with a diagnosis of unstable angina. 2. Patients who refused angiography or passed away before performing angiography.

Statistical analysis methodology: The statistical program SPSS 20.0 (Statistical Package for Social Sciences) was used to analyze the data. The Student’s t-test and the Wilcoxon test were used to compare continuous variables, as appropriate, while the Chi-square test was used to compare categorical variables. Categorical variables were presented according to their absolute and relative frequency expressed in percentage. The statistical test of one-way ANOVA was used to determine whether there were any statistically significant differences between the means of three or more independent (unrelated) groups. Continuous data are presented with mean (M) and standard deviation (SD). Statistical significance is defined for *p* ≤ 0.05. Tables were used to visualize the data.

## 3. Results

There were 334 patients included, 241 OMI and 93 NOMI. Of the patients, 98 (29.3%) were STEMI(−)OMI, 73 (21.9%) were STEMI(−)NOMI, 143 (42.8%) were STEMI(+)OMI, and 20 (6.0%) were STEMI(+)NOMI (see Table 1) and their clinical characteristics are shown in Table 2 (See Table 2). Only 15 patients (11%) of STEMI(−)OMI had PCI performed within the first 12 h, vs. 110 patients (77%) of STEMI(+)OMI, *p* < 0.001 (see Table 3). There was no difference in the percent of patients requiring PCI between the STEMI(+)OMI 133 patients (93%) and STEMI(−)OMI 87 patient (89%) groups (*p* < 0.001) (see Table 4). The average of the highest days of stay resulted in the group of STEMI(+)NOMI pa-tients with 9.15 days(See Table 5).

Average EF was: STEMI(+)NOMI 44.70%, STEMI(−)NOMI 50.47%, STEMI(−)OMI 48.70%, STEMI(+)OMI 42.87%, *p* < 0.001 (see Table 6). The mortality was 19 patients (5.7%), 14 (4.2%) STEMI(+)OMI, 2 (0.6%) STEMI(+)NOMI, 3 (0.9%) STEMI(−)OMI, 0% STEMI(−)NOMI, *p* = 0.013 (see Table 7). The mechanical complications were present in 58 patients (46.8%) with STEMI(+)OMI and in 39 patients (46.4%) with STEMI(−)OMI. In addition, 23 patients (18.5%) with STEMI(+)OMI and 11 patients (13.1%) with STEMI(−)OMI developed electrical complications (see Table 8).

In total, 334 patients were included, of whom 241 patients (72.2%) had OMI and 93 (27.8%) had NOMI. In addition, 98 patients (29.3%) were STEMI(−)OMI, 73 patients (21.9%) were STEMI(−)NOMI, 143 patients (42.8%) were STEMI(+)OMI, and 20 patients (6.0%) were STEMI(+) NOMI.

In total, 19 patients (5.7%) died, 14 patients (4.2%) were STEMI(+)OMI, 2 patients (0.6%) were STEMI(+)NOMI, 3 patients (0.9%) were STEMI(−)OMI, and 0 patient were STEMI(−)NOMI. Of the patients, 14 (10%) from the STEMI(+)OMI group and 3 (3%) from the STEMI(−)OMI group died.

**Figure 2:**
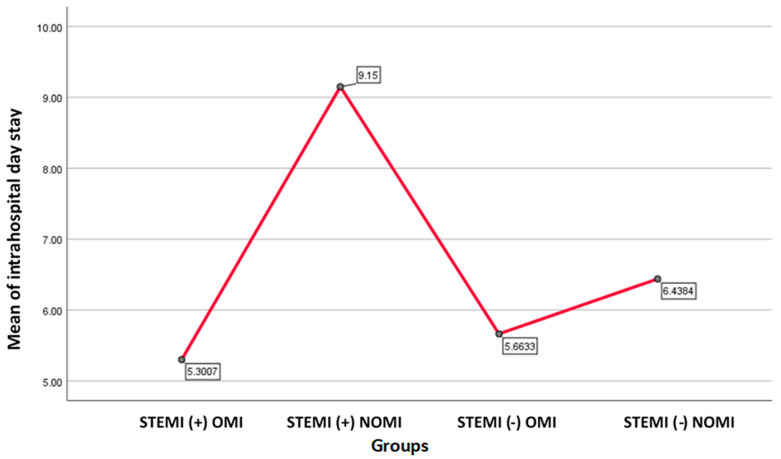
Graph showing mean of hospital day stay for each group. STEMI(+)NOMI had the highest hospital day stay, followed by STEMI(−)NOMI.

The average of the highest days of stay resulted in the group of STEMI(+)NOMI patients with 9.15 days, followed by STEMI(−)NOMI with 6.44 days, STEMI(−)OMI patients with 5.66 days, and STEMI(+)OMI with 5.30 days (one-way ANOVA F = 4.495, *p* = 0.004) [Figure 2].

**Figure 3:**
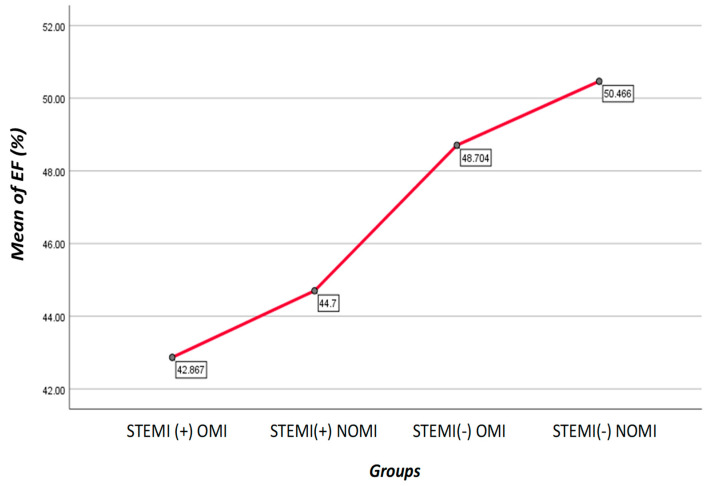
Graph shows mean of EF(%) for each group. Patients with STEMI(−)NOMI had the highest EF, while the patients with STEMI(+)OMI had the lowest EF.

Average EF was: STEMI(+)NOMI 44.70%, STEMI(−)NOMI 50.47%, STEMI(−)OMI 48.70%, and STEMI(+)OMI 42.87%, *p* < 0.001 [Figure 3].

In total, 110 patients (77%) of STEMI(+)OMI patients had PCI performed within the first 12 h, vs. 15 patients (11%) of STEMI(−)OMI. In addition, 125 (87.4%) STEMI(+)OMI patients performed PCI within 24 h vs. 41 patients (42.7%) in the STEMI(−)OMI group. Of the majority of patients belonging to the STEMI(−)OMI group, 55 (57.3%) underwent PCI after 24 h, and 34 patients (61.7%) underwent PCI after 48 h.

The group with fewer complications was STEMI(−)NOMI, with 29 patients (44.6%). In total, 101 patients (34.6% of patients) had no complications. We found that 134 patients (45.9%) had only mechanical complications, whereas STEMI(+)OMI and STEMI(−)OMI had a similar frequency of complications. The mechanical complications were present in 58 patients (46.8%) with STEMI(+)OMI and in 39 patients (46.4%) with STEMI(−)OMI. In addition, 23 patients (18.5%) of the STEMI(+)OMI and 11 patients (13.1%) of the STEMI(−)OMI developed electrical complications. Patients who had electrical and mechanical complications made up the smallest group, with only 13 patients (4.5% of the total).

There is no difference in the percentage of patients requiring PCI between the STEMI(+)OMI 133 patients (93%) and STEMI(−)OMI 87 patients (89%) groups.

## 4. Discussion

Contemporary articles display the same essence: “The STEMI criteria underestimate many patients with the same risk as STEMI ones” [3,4,5,6,7,8,11,12]. Our results support the idea that the STEMI criteria fail to diagnose a large proportion of OMI and fail to identify patients who require emergent reperfusion and who will have complications, such as STEMI(−)OMI patients with the same pathology and risk as STEMI(+)OMI patients.

For years, authors have attempted to compensate for this discrepancy with terminologies such as “STEMI equivalents”, “Subtle STEMI”, or “Semi-STEMI”. Nevertheless, only 11 patients (11%) of patients with STEMI(−)OMI received reperfusion therapy within 12 h in our cohort. The terminology “STEMI” suggests that what is important is ST elevation, when in reality it is the acute coronary occlusion that is important [3,7,8].

ESC 2023 Guidelines for the Management of Acute Coronary Syndromes in Patients Presenting without Persistent ST-Segment Elevation emphasize early risk stratification, dual antiplatelet therapy (DAPT), and optimal timing for invasive strategies. Key updates include guidance on P2Y12 inhibitors, routine coronary angiography, and tailored approaches for high-risk groups, including the elderly and those with renal impairment [13].

In their trial, Jolly, S.S., & Mehta, S.R. investigated the optimal timing for coronary intervention in patients with NSTEMI. It indicates that early intervention within 24 h of symptom onset can benefit high-risk patients, while a delayed approach may be appropriate for those at lower risk [14].

The ESC guidelines provide specific recommendations for revascularization in patients presenting with STEMI [15]. Firstly, reperfusion therapy is recommended for all patients diagnosed with STEMI, characterized by persistent ST-segment elevation or its equivalents, and with symptoms lasting less than 12 h (Class IA) [15]. For patients with STEMI symptoms lasting more than 12 h, primary PCI is recommended if symptoms persist, if there is hemodynamic instability, or if life-threatening arrhythmias occur (Class IC) [15]. Additionally, primary PCI should be considered for patients who present late, within 12 to 48 h after symptom onset (Class IIaB) [15]. However, routine PCI of an occluded infarct-related artery is not recommended for patients who present more than 48 h after symptom onset and who do not have persistent symptoms (Class IIIA) [15].

The 2017 ESC guidelines on STEMI also highlight that in certain situations, patients may have coronary artery occlusion or global ischemia without the characteristic ST-segment elevation [2]. Such situations include left bundle branch block, hyperacute T-waves, isolated ST-segment depressions in anterior leads, ventricular pacing, and universal ST-segment depressions with ST elevation in aVR. In patients with these ECG changes and a clinical presentation suggestive of myocardial ischemia, a strategy of primary PCI (urgent angiography and PCI if indicated) should be implemented [2].

Furthermore, a meta-analysis from de Alencar Neto et al. (2024) focuses on the diagnostic accuracy of ST-segment elevation in identifying acute coronary occlusion, differentiating true STEMI cases from mimics. It emphasizes the importance of distinguishing STEMI-like ECG patterns, such as early repolarization, pericarditis, and hyperkalemia, which can present similarly but require different management [3].

Sgarbossa et al. in their study evaluate the use of high-sensitivity troponins to improve the diagnostic accuracy in patients presenting with STEMI-like ECG patterns. The study found that elevated troponin levels in the absence of true coronary occlusion were often associated with conditions like myocarditis, which necessitate different therapeutic approaches [16].

Thus, in our cohort, “STEMI” and “ST-Elevation” were obstacles to improving the management of AMI in 96 patients (40% of our OMI patients). When we see an ECG in the setting of potential ischemia, we should first look for any pattern that reliably predicts OMI, because these are the patients who benefit from emergency reperfusion [3,4,5,6,7,8,11]. In addition to ischemic ST elevation, we must consider minimal ST elevations that do not meet the classic criteria, hyperacute T waves (including the de Winter pattern), reciprocal ST depressions and/or hyperacute negative T waves, depressions of ST in V1–V4 indicative of posterior AMI, acute pathological Q waves (Q waves associated with minimal ST elevation, not attributable to a previous myocardial infarction), terminal QRS distortion (loss of the preceding S wave in the context of minimal ST elevation), any inferior ST elevation with any ST depression or T wave inversion in aVL, and modified Sgarbossa criteria for patients with LBBB or paced rhythm [3,4,5,6,7,8,11,12].

In their study, Aslanger et al. found that ”The Aslanger Pattern” was identified in a significant portion of the study population. Specifically, it was observed in about 4–6% of patients with inferior STEMI. They concluded that the “Aslanger Pattern”, seen in patients with myocardial infarction, helps identify those with acute coronary occlusion (ACO), particularly in cases of inferior MI without classic ST-elevation. The Aslanger pattern involves subtle ST changes in the inferior and anterior leads, which suggest an occlusion of the circumflex or right coronary artery. The study compared the Aslanger pattern with other established diagnostic patterns and found it to be a useful addition to the diagnostic toolkit for identifying inferior STEMI. The results underscore the value of incorporating the Aslanger pattern into routine ECG interpretation to improve diagnostic accuracy for inferior STEMI [17,18].

Table 9 presents ECG patterns that do not meet the criteria for STEMI but are found to be OMI.

Lindow et al. included 623 patients in their article, among whom 441 (71%) had LBBB and 182 (29%) had VPR (ventricular paced rhythm) 82 (13%) of these patients were diagnosed with AMI, and an OMI was identified in 15 (2.4%) cases. Sensitivity/specificity of the original unweighted Sgarbossa criteria were 26.7/86.2%, for Barcelona criteria 53.3/82.2%, for MSC 60.0/86.0%, and for Selvester criteria 46.7/88.3%. In this setting with a low prevalence of OMI, positive predictive values were low (Sgarbossa: 4.6%; MSC (modified Sgarbossa criteria): 9.4%; Barcelona criteria: 6.9%; Selvester criteria: 9.0%) and negative predictive values were high (all >98.0%). Their results suggest that ECG criteria alone are insufficient in predicting the presence of OMI in an ED setting with a low prevalence of OMI, and the search for better rapid diagnostic instruments in this setting should continue [12]. Recently, a deep neural network has been shown to diagnose OMI with twice the sensitivity of STEMI criteria at the same specificity [11].

It is important to highlight the prognostic stratification in STEMI cases. Recent data highlight the necessity of multiparametric risk stratification, including ECG and imaging parameters [34]. In their study, Bergamaschi et al. explored how the non-infarcted myocardium and the surrounding tissue respond acutely after a STEMI (ST-elevation myocardial infarction). It was identified that even the non-infarcted myocardium shows significant changes in T2 values, indicating the presence of edema and possible inflammatory responses in areas not directly affected by the heart attack. Alterations in T2 values suggested that STEMI causes a broader impact on the heart, not just limited to the area of infarction. These findings suggest that post-STEMI treatment should consider the entire heart, as remote myocardial regions and non-infarcted areas also undergo significant physiological changes, potentially affecting patient outcomes. T2 mapping proves to be a valuable tool for evaluating myocardial changes beyond the infarct zone, offering insights into the broader impact of STEMI on the heart and aiding in the development of more comprehensive therapeutic strategies [34].

Recently, a review article thoroughly examined the preclinical and clinical evidence regarding the use of SGLT2 inhibitors as adjuncts to standard care in acute coronary syndrome (ACS) patients. The article highlights that preclinical studies suggest these inhibitors may reduce ischemia-reperfusion injury and myocardial infarct size, especially with prior treatment. Clinical trials and real-world data also indicate potential benefits in acute ischemic settings, including improved left ventricular function, decongestion, and cardiometabolic parameters. However, further studies are needed to assess the effectiveness of SGLT2 inhibitors in STEMI(+)/(−) and OMI/NOMI populations [35].

It is important to highlight that the new AMI classification will face implementation challenges. A paradigm shift requires changes in the AMI management protocols and this may lead to potential confusion among the involved medical staff. Therefore, in such conditions, multidisciplinary training, seminars, or lectures will be continuously required. In this way, these protocols will be understood and implemented easily.

On the other hand, since the current paradigm excludes 40% of OMI from immediate revascularization, we will need greater capacities and more human resources to cope with 40% more immediate revascularizations.

## 5. Study’s Limitations

First, the study is a retrospective analysis conducted on 334 patients and all cases were obtained from a single tertiary center that offers emergency coronary angiography service (24/7). Therefore, despite the small sample size and the length of follow-up, this study suffers from the basic limitations of a retrospective registry, and these conclusions should therefore be confirmed in a randomized study.

Second, data on chest pain onset times were obtained from the patient’s cardiology referral note on the chart. Third, of the 375 patients who were initially identified, 41 patients were excluded because they refused PCI or passed away before performing PCI. Fourth, among the 334 included patients, data on 42 patients were missing, displaying potential complications. Thus, not fulfilling at the end the wished statistical significance due to the restricted number of events.

## 6. Conclusions

STEMI(−)OMI patients had significant delays in catheterization, and had complications similar to STEMI(+)OMI. These data add further support to refocusing the paradigm of acute MI to improve recognition and rapid reperfusion of all OMIs, rather than only those with STEMI criteria. Clinicians should keep in mind that OMI is a clinical diagnosis, which includes OMI ECG findings but also clinical parameters, and is not defined entirely by the ECG or angiogram. The critical next steps of this paradigm shift include finding reliable methods to quickly identify all OMI patients for emergent reperfusion.

## Figures and Tables

**Table 1:** The results of the OMI/NOMI classification.

	OMI or NOMI		Total
	OMI	NOMI	
STEMI	143	20	163
	42.8%	6.0%	48.8%
NSTEMI	98	73	171
	29.3%	21.9%	51.2%
Total	241	93	334
	72.2%	27.8%	100.0%

NSTEMI = non-ST-segment elevation Miocardial infarction. STEMI = ST-segment elevation Myocardial infarction.

**Table 2:** Clinical characteristics of all patients in each subgroup.

Characteristic	STEMI(+)OMI n = 143	STEMI(+)NOMI n = 20	STEMI(−)OMI n = 98	STEMI(−)NOMI n = 73
Age, y, mean (SD)	65.06 (13.33)	59.16 (12.43)	64.31 (10.43)	68.94 (11.78)
Female, n (%)	41 (28.6%)	5 (25%)	22 (22.4%	29 (39.7%)
Known CAD, n (%)	9 (6.5%)	0 (0%)	9 (9.1%)	8 (10.9%)
Prior CABG, n (%)	2 (1.3%)	0 (0%)	5 (5.1%)	3 (4.1%)
Prior CVA, n (%)	2 (1.3%)	1 (5%)	0 (0%)	3 (4.1%)
CKD, n (%)	11 (7.7%)	1 (5%)	5 (5.1%)	7 (9.5%)
CHF, n (%)	56 (39.1%)	6 (30%)	64 (65.9%)	49 (67.1%)
Diabetes, type 2, n (%)	16 (11.2%)	6 (30%)	42 (42.8%)	38 (52%)
IGT, n (%)	3 (2%)	1 (5%)	5 (5.1%)	2 (2.7%)
HLD, n (%)	100 (70%)	8 (40%)	88 (89.7%)	54 (73.9%)
HTN, n (%)	108 (75.5%)	10 (50%)	88 (89.7%)	50 (68.4%)
PVD, n (%)	3 (2%)	0 (0%)	2 (2%)	0 (0%)

CABG = coronary artery bypass grafting; CAD = coronary artery disease; CKD = chronic kidney disease; CVA = cerebrovascular accident; IGT = impaired glucose tolerance; HLD = hyperlipidemia; HTN = hypertension; NOMI = non-occlusion MI; OMI = occlusion Mi; PVD = peripheral vascular disease; SD = standard deviation; STEMI = ST-segment elevation myocardial infarction.

**Table 3:** Total ischemic time in each of the groups.

Time Interval (hours)	Groups				Total
	STEMI(+)OMI	STEMI(+)NOMI	STEMI(−)OMI	STEMI(−)NOMI	
0–2	7	0	1	0	8
	4.9%	0.0%	1.0%	0.0%	2.4%
2–6	52	5	2	2	61
	36.4%	25.0%	2.1%	2.8%	18.4%
6–12	51	7	12	6	76
	35.7%	35.0%	12.5%	8.3%	23.0%
12–24	15	2	26	12	55
	10.5%	10.0%	27.1%	16.7%	16.6%
24–48	3	1	21	15	40
	2.1%	5.0%	21.9%	19.4%	11.8%
>48	15	5	36	38	94
	10.5%	25.0%	35.4%	52.8%	27.8%
Total	143	20	98	73	334
	100.0%	100.0%	100.0%	100.0%	100.0%
Median ischemic time	8.0000	10.0000	48.0000	72.0000	
IQR	7.00	113.75	48.00	96.00	

χ^2^ = 145.89, *p* < 0.001.

**Table 4:** STEMI(+)OMI vs. STEMI(−)OMI interventions.

Interventions	STEMI(+)OMI	STEMI(−)OMI	STEMI(+)NOMI	STEMI(−)NOMI
Angiogram	143 (100%)	98(100%)	20 (100%)	73(100%)
PCI	134 (93%)	87 (88.7%)	0 (0%)	61 (83.6%)
CABG *	5 (3.4%)	6 (6.2%)	0 (0%)	0 (0%)
PCI + CABG †	4 (2.6%)	5 (5.1%)	0 (0%)	9 (12.4%)
Total	143 (100%)	98 (100%)	20 (100%)	73 (100%)

*χ*^2^ = 14.390, *p* = 0.496. * Urgent coronary artery bypass grafting (CABG). † PCI followed by CABG in a second moment.

**Table 5:** Average hospital length of stay in each group.

	N	Mean (day)	SD	95% Confidence Interval for Mean		Minimum (day)	Maximum (day)
STEMI(+)OMI	143	5.30	3.95	4.65	5.95	1.00	30.00
STEMI(+)NOMI	20	9.15	13.22	2.96	15.34	1.00	64.00
STEMI(−)OMI	98	5.66	3.08	5.05	6.28	0.00	19.00
STEMI(−)NOMI	73	6.44	2.92	5.76	7.12	2.00	17.00
Total	334	5.89	4.70	5.38	6.39	0.00	64.00

One-way ANOVA, F = 4495, *p* = 0.004. STEMI(+) = ST-segment elevation criteria myocardial infraction criteria fulfilled. STEMI(−) = ST-segment elevation myocardial infraction criteria not fulfilled.

**Table 6:** Ejection fraction in each of the groups.

	N	Mean	SD	95% Confidence Interval for Mean
STEMI(+)OMI	143	42.9	9.52	42.87 ± 1.58
STEMI(+)NOMI	20	44.7	11.11	44.70 ± 5.2
STEMI(−)OMI	98	48.7	12.00	48.70 ± 2.4
STEMI(−)NOMI	73	50.5	10.77	50.47 ± 2.51
Total	334	46.4	11.13	46.355 ± 1.22

One-way ANOVA, F = 20,630, *p* < 0.001.

**Table 7:** In-hospital mortality after PCI in each of the groups.

Exitus	Groups				Total
	STEMI + OMI	STEMI + NOMI	STEMI − OMI	STEMI − NOMI	
Yes	14 4.2%	2 0.6%	3 0.9%	0 0%	19 5.7%
Total	143	20	98	73	334

χ^2^ = 10.84, *p* = 0.013. STEMI(+) = ST-segment elevation criteria myocardial infraction criteria fulfilled. STEMI(−) = ST-segment elevation myocardial infraction criteria not fulfilled.

**Table 8:** The balance of intrahospital complications in each of the groups.

Complications	Groups				Total
	STEMI(+)OMI	STEMI(+)NOMI	STEMI(−)OMI	STEMI(−)NOMI	
None	36	3	33	29	101
	29.0%	15.8%	39.3%	44.6%	34.6%
* Mechanical complications	58	11	39	26	134
	46.8%	57.9%	46.4%	40.0%	45.9%
† Electrical complications	23	3	11	7	44
	18.5%	15.8%	13.1%	10.8%	15.1%
Mechanical and electrical complications	7	2	1	3	13
	5.6%	10.5%	1.2%	4.6%	4.5%
Total	124	19	84	65	292
	100.0%	100.0%	100.0%	100.0%	100.0%

* As mechanical complications we have included acute ventricular failure, cardiogenic shock, rupture of the interventricular septum, rupture of the free wall, rupture of the papillary muscle, and pericarditis. † As electrical complications we have included ventricular, supraventricular arrhythmias, and atrioventricular and interventricular blocks.

**Table 9:** STEMI(−)OMI patterns.

STEMI(−)OMI Patterns
LBBB or paced rhythm meeting Sgarbossa criteria [16,19].
2. Terminal QRS distortion (subtle STE) [20].
3. De Winter/hyperacute T-waves [21,22,23].
4. ST depressions in V1–4, without changes in V5–6 and without ST-segment elevation in V7–9 (Isolated posterior MI) [24,25].
5. Diffuse ST-segment depressions with ST-segment elevation in aVR [26].
6. New bifascicular block (RBBB + LAFB) or new LBBB [27,28,29].
7. Wellens syndrome [30,31,32,33].
8. Aslanger pattern: ST-segment elevation in lead III with reciprocal depression in aVL [17,18].

## Data Availability

The original contributions presented in the study are included in the article, further inquiries can be directed to the corresponding author.
